# Abemaciclib treatment patterns and outcome in HR+/HER2- locally advanced or metastatic breast cancer: a real-world study from Kuwait and Lebanon

**DOI:** 10.3389/fonc.2025.1437380

**Published:** 2025-06-17

**Authors:** Anwar Al Nouri, Faisal Al Terkait, Nagi El Saghir, Hady Ghanem, Joseph Kattan, Cynthia Bishouti, Jade Dreyer, Soniya Rai, Alexandru Rosca

**Affiliations:** ^1^ Medical Oncology Department, Kuwait Cancer Control Center, Kuwait, Kuwait; ^2^ Division of Hematology/Oncology, American University of Beirut Medical Center (AUBMC), Beirut, Lebanon; ^3^ Lebanese American University (LAU) Medical Center-Rizk Hospital (LAUMC-RH), Beirut, Lebanon; ^4^ Department of Hematology-Oncology, Hotel Dieu De France, Beirut, Lebanon; ^5^ Oncology Medical Affairs, Eli Lilly U.A.E. (Suisse) SA (United Arab Emirates Division), Dubai, United Arab Emirates

**Keywords:** breast neoplasms, disease-free survival, CDK inhibitor, abemaciclib, metastatic breast cancer

## Abstract

**Purpose:**

The burden of breast cancer is still growing in the Middle East and North Africa (MENA). BC patients typically present with more advanced stages than in Western countries. Limited information is available regarding the safety and efficacy of novel molecules for advanced BC in the Middle East region. The present real-world study evaluated the treatment patterns and survival outcomes of abemaciclib in patients with hormone receptor-positive (HR+)/human epidermal growth factor receptor-2-negative (HER2-) locally advanced or metastatic BC (mBC) in Kuwait and Lebanon.

**Methods:**

The TRACE study is an observational, retrospective, multicenter, single-arm cohort study. Medical records of HR+/HER2- locally advanced or mBC women were retrieved if they received abemaciclib as part of their treatment in Kuwait and Lebanon. Only patients who received abemaciclib monotherapy or in combination with other treatments for at least three months before data collection were included.

**Results:**

Eighty-five patients met the eligibility criteria (Kuwait =42 patients, Lebanon =43). Nearly 57% of the patients received abemaciclib in the first-line setting, 19.8% received it in the second-line, and 16.5% received it at third or later lines of treatment. Abemaciclib 150mg twice daily was administered in combination with other treatments, mainly endocrine therapy, in 95.3% of the patients. Overall, 18 patients (21.4%) had a dose reduction at the end of the third month of abemaciclib treatment. After three months of treatment, the rates of complete response (CR) and partial response (PR) as the best response were 6.9% and 63.8%, respectively, with an objective response rate (ORR) of 70.7%. The 12-month progression-free survival (PFS) was 33.3% in the monotherapy group and 79.6% in the combination group.

**Conclusion:**

The present real-world evidence confirms the feasibility and effectiveness, in terms of response rate and PFS, of abemaciclib in patients with HR+/HER2- patients with locally advanced or mBC from Kuwait and Lebanon in the Middle East region.

## Introduction

According to the 2020 GLOBOCAN statistics, breast cancer (BC) has become the most common cancer type worldwide, accounting for 11.7% of new cancer cases and 6.9% of cancer-related deaths (1). BC is a heterogeneous disease in which the expression levels of surface and nuclear receptors are the main biomarkers of BC subtypes, prognosis, treatment, and patient outcomes (2). Nearly two-thirds of BC patients are hormone receptor-positive (HR+)/human epidermal growth factor receptor-2-negative (HER2-) (3). In locally advanced and metastatic BC (mBC) settings -collectively termed advanced BC- the treatment selection is based on subtype, history of prior treatment, and menopausal status. International guidelines recommend endocrine therapy (ET) for patients with HR+/HER2- locally advanced or mBC. Chemotherapy is used for patients with ET-resistant disease (4). Until recently, and despite the generally favorable prognosis of HR+/HER2- BC, patients with advanced or metastatic diseases still showed suboptimal survival (5, 6). Besides, limited options with modest efficacy are available for cases refractory to ET (7).

The introduction of molecular targeted therapy has revolutionized the treatment landscape and improved the outcomes of different BC subtypes, including HR+/HER2- locally advanced or mBC (8). Patients with HR+ BC usually show cyclin D overexpression, which promotes tumor cell proliferation, cell growth, anti-apoptotic activities, and genome instability via upstream activation of cyclin-dependent kinases (CDK) (9). Several CDK were identified, including CDK4 and CDK6, which were found to be involved in tumor cell proliferation in HR+ BC (10). Preclinical investigations and early clinical trials evaluated selective CDK 4/6 inhibitors in HR+ BC and showed promising antitumor activities and a well-tolerable safety profile (10–12).

Abemaciclib is an orally potent selective CDK 4/6 inhibitor that significantly improved the survival outcomes of HR+/HER2- patients with advanced BC in the first and second-line settings. In the final results of MONARCH 1 trial at 18 months, single-agent abemaciclib led to median progression-free survival (PFS) and OS of 6 and 22.3 months, respectively, with a disease control rate (DCR) of 67.4%, in refractory HR+/HER2- mBC (13). The benefits of abemaciclib were also evident in MONARCH 2, which recruited HR+/HER2- locally advanced or mBC progressed on ET. The abemaciclib plus fulvestrant had a median OS of 45.8 months (14). In the pivotal MONARCH 3 trial, abemaciclib plus ET was associated with a significantly longer PFS and increased objective response rate (ORR) than ET in treatment-naïve patients with HR+/HER2- locally advanced or mBC (15). In return, abemaciclib combined with ET is approved for treatment-naïve or pretreated women with HR+/HER2- advanced BC. At the same time, it is the only approved CDK4/6 inhibitor as a single agent for women with mBC who progressed on ET or prior chemotherapy (6). Recently, abemaciclib combined with ET was approved as adjuvant therapy for high-risk early BC (16).

The burden of breast cancer (BC) is still growing in the Middle East and North Africa (MENA). Previous reports estimated that the MENA region had witnessed the largest increase in BC incidence over the past three decades (17, 18). Data show that BC patients from the region are often diagnosed at younger ages [50% below age 50 (19)], with more aggressive clinicopathologic patterns and more advanced stages than in Western countries (20). Modern management, including CDK4/6 inhibitors, is widely practiced in many centers in the Middle East. Still, limited information is available regarding the safety and efficacy of CDK 4/6 inhibitors in the region. There is an unmet clinical need to explore the treatment patterns and outcomes of novel targeted therapy in patients with advanced BC from the MENA. Therefore, the present real-world study evaluated the treatment patterns and survival outcomes of abemaciclib in patients with HR+/HER2- locally advanced or mBC in two Middle Eastern countries, Kuwait and Lebanon, which have one of the highest incidences of BC in the region (21, 22).

## Patients and methods

The present report was prepared in concordance with the STROBE statement (**Supplementary Table 1**) (23). All study procedures adhered to the principles of the latest version of the Declaration of Helsinki (24) and applicable local regulatory laws. The study was approved by the local ethics committee of each participating center.

### Study design, participants, and setting

The TRACE was an observational, retrospective, multicenter, single-arm cohort study. Medical records of HR+/HER2- locally advanced or mBC women were retrieved if they received abemaciclib as a part of routine clinical practice in Kuwait and Lebanon. Adult women (age ≥18 years old) were deemed eligible if they had a confirmed diagnosis of locally advanced or mBC, whether *de-novo* or progressed/recurred from early BC, histologically proven HR+/HER2- status, and received abemaciclib monotherapy or in combination with other treatments for at least three months before data collection. Eligible patients received abemaciclib at the discretion of the treating physicians only. There was no attempt to influence the prescribing patterns of any individual investigator. We excluded patients who received abemaciclib in an adjuvant setting, women who received abemaciclib as a part of a clinical trial, and/or patients who had concurrent primary malignancy.

Consecutive records of eligible patients were retrieved from three centers in Lebanon and one center in Kuwait. The data collection covered the period from the date of approval of abemaciclib in each country (June 2018 in Kuwait and January 2020 in Lebanon) to September 2022. Data covering at least three months ±30 days after initiating abemaciclib were retrieved.

### Data collection

The following data were retrieved from the medical records of eligible patients: demographic characteristics, anthropometric measures, family history of cancer, associated comorbidities, disease characteristics (age at diagnosis of locally advanced or mBC, stage at diagnosis, Eastern Cooperative Oncology Group [ECOG] performance status [PS], and number and sites of metastasis), treatment patterns (age at abemaciclib initiation, dose, frequency, line of therapy, need for dose reduction, concomitant medications, and co-current radiotherapy or surgery), date of progression or death on abemaciclib line of therapy, date of last follow-up, and the recorded best response. The best response was assessed using the Response Evaluation Criteria in Solid Tumors (RECIST) version 1.1.

### Study endpoints

The primary endpoint of the present study was to describe the treatment patterns of abemaciclib monotherapy or in combination with other treatments for patients with HR+/HER2- locally advanced or mBC in Kuwait and Lebanon. The secondary outcomes were to describe the demographic and clinical characteristics of patients who were initiated on abemaciclib in both countries, PFS on abemaciclib, the best response on abemaciclib according to RECIST 1.1, and objective response rate (ORR), defined as the rate of patients who achieved complete response (CR) or partial response (PR).

### Sample size calculation

The study was descriptive, with no formal sample size computation needed. However, with 100 participants, the study would be able to estimate the prevalence of any binary outcome within a margin of error of at most ±10% using a 95% confidence interval (CI). The study recruited 85 participants, which achieved a precision of 9% in detecting a prevalence of 25% for any binary outcomes within a margin of error of at most ±10% using a 95% confidence interval (CI).

### Statistical analysis

All analyses were done using IBM SPSS Statistics for Windows (Version 27.0). Descriptive analysis was conducted using the mean ± standard deviation (SD) and counts for continuous and categorical variables, respectively. The best response rate was calculated from the total number of patients with medical records to extract the RECIST 1.1 response. The Kaplan- Meier curves were used to calculate the PFS and OS of the study cohort, which was stratified into monotherapy and combination groups. The PFS and OS were defined as the time from initiating abemaciclib to disease progression/death and death, respectively. Subgroup analyses for each country (Kuwait and Lebanon) were conducted. Besides, the treatment patterns, survival outcomes, and response rates were calculated at three timepoints: at the start of abemaciclib, whether as a single-agent treatment or combination treatment (1^st^ timepoint); at the end of three months (± 1 month) of abemaciclib treatment (2^nd^ timepoint); and at the earliest of death, the last medical record entry, or the end of the observation period “defined as the date of data abstraction” (3^rd^ timepoint).

## Results

**Figure 1:**
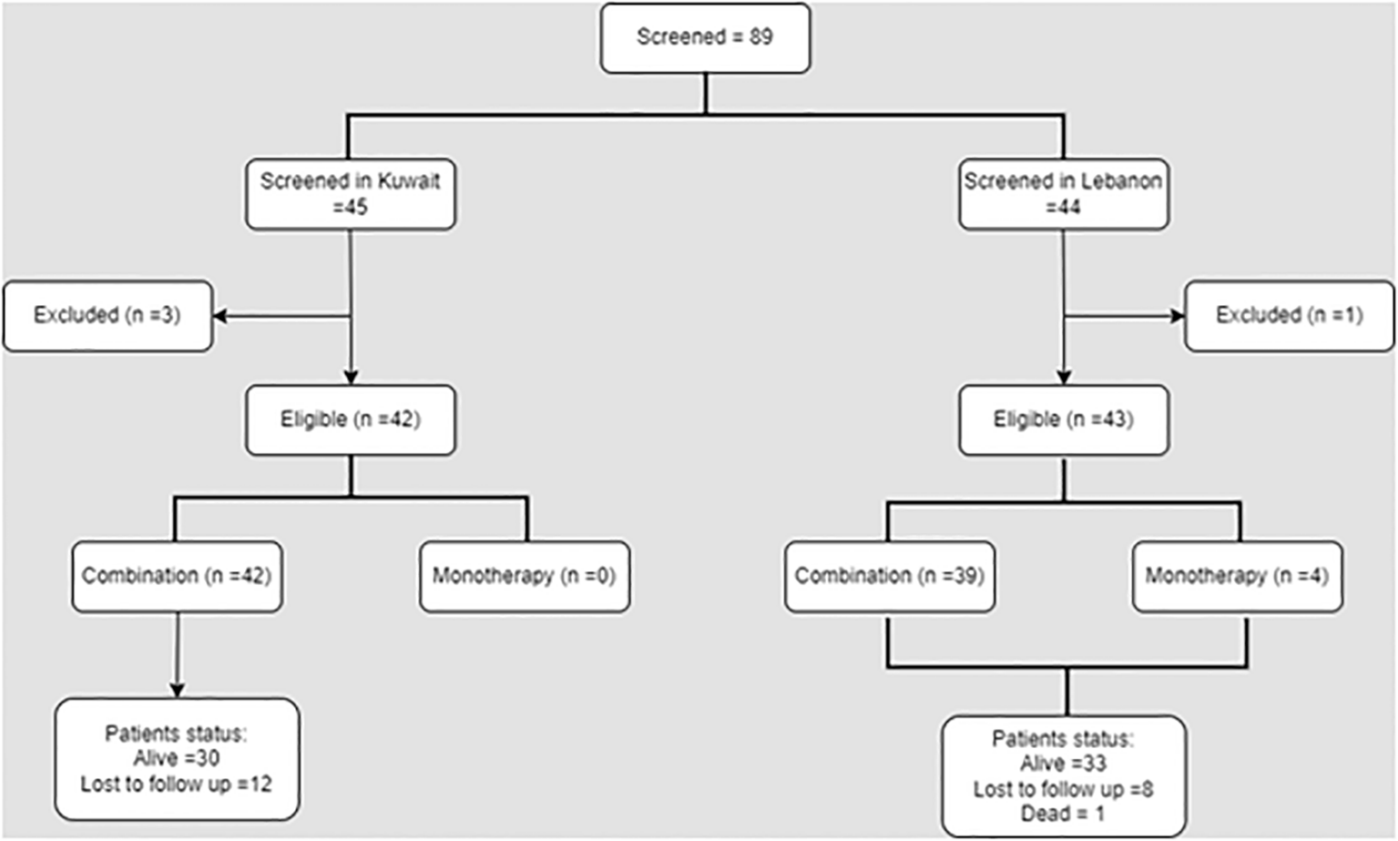
Study flowchart.

Out of the 89 patients available during the data collection period, 85 met the eligibility criteria and were retrieved in the present study. Forty-two eligible patients were from Kuwait and received abemaciclib in combination with other treatments. On the other hand, out of the 43 patients from Lebanon, four patients received abemaciclib monotherapy (**Figure 1**).

**Table 1** shows the demographics and baseline characteristics of the patients at the time of the start of abemaciclib. The mean age of the included patients was 55.6 ± 12.1 years, with a slightly higher average in Lebanon (mean age =58.0 ± 12.6 years). Overall, 57.6% of patients were post-menopausal, and 35.3% had a family history of cancer. Regarding anthropometric measures, the mean body mass index (BMI) of the included patients was 28.5 ± 5.9Kg/m^2^, with a slightly higher average BMI in Kuwait. Nearly 73% of the patients with available ECOG PS had ECOG PS 0-1. Most patients had mBC (85.9%) at the time of abemaciclib initiation, while the mean age at the diagnosis of locally advanced or metastatic disease was 52.4 ± 11.9 years. The average number of metastatic sites was 2.1 ± 1.1 sites, and bone was the most common metastatic site (present in around 60% of cases) in the study’s cohort. Around one-third of patients had liver or lung metastases.

**Table 1:** Patient demographics and baseline characteristics at abemaciclib initiation.

Variables	Total (N=85)	Kuwait (N=42)	Lebanon (N=43)
**Age (years), mean (SD)**	55.6 (12.1)	53.1 (11.1)	58.0 (12.6)
Menopausal Status, No. (%)			
Pre-Menopause	25 (29.4%)	19 (45.2%)	6 (14%)
Post-Menopause	49 (57.6%)	23 (54.8%)	26 (60.5%)
Not Available	11 (12.9%)	0 (0%)	11 (25.6%)
**Height (cm), mean (SD)**	(n =58)	(n =36)	(n =22)
	158.3 (6.0)	158.1 (5.4)	158.7 (7.0)
**Body Mass Index (kg/m^2^), mean (SD)**	28.5 (5.9)	29.2 (5)	27.4 (7.1)
**Family history of cancer, No. (%)**	30 (35.3%)	17 (40.5%)	13 (30.2%)
**ECOG Status, No. (%)**	(n =59)	(n= 42)	(n =17)
0	18 (30.5%)	6 (14.3%)	12 (70.6%)
1	25 (43.1%)	22 (52.4%)	3 (17.6%)
2	13 (15.3%)	11 (26.2%)	2 (11.8%)
3	3 (22.1%)	3 (7.1%)	0 (0.0%)
4	0 (0%)	0 (0%)	0 (0.0%)
Patient Diagnosis, No. (%)			
Locally Advanced Breast Cancer	12 (14.1%)	2 (4.8%)	10 (23.3%)
Metastatic Breast Cancer	73 (85.9%)	40 (95.2%)	33 (76.7%)
**Age at Diagnosis (Years), mean (SD)**	(n =79)	(n =40)	(n =39)
	52.4 (11.9)	51.4 (10.9)	53.3 (12.9)
Stage, No. (%)			
Stage IIIb	3 (3.5%)	0 (0%)	3(7%)
Stage IIIc	3 (3.5%)	2 (4.8%)	1(2.3%)
Stage IV	70 (82.4%)	39 (92.9%)	31(72.1%)
Unknown/Not Assessed/Not Available	9 (10.6%)	1 (2.4%)	8 (18.6%)
**Number of Metastatic Sites, mean (SD)**	2.1 (1.1)	2.1 (1.1)	2.1 (1.2)
Sites of Metastasis, No. (%)			
Bones	46 (54.1%)	26 (61.9%)	18 (54.5%)
Lymph nodes-regional	23 (27.1%)	11 (26.2%)	12 (27.9%)
Lymph nodes-distal	9 (10.6%)	5 (11.9%)	4 (12.1%)
Lungs	24 (28.2%)	15 (35.7%)	9 (27.3%)
Liver	25 (29.4%)	15 (35.7%)	10 (30.3%)
Others	14 (16.5%)	5 (11.9%)	9 (20.9%)
**Radiotherapy before Abemaciclib, No. (%)**	35 (41.2%)	11 (26.2%)	24 (57.1%)

SD, Standard deviation; ECOG, Eastern Cooperative Oncology Group.

**Figure 2:**
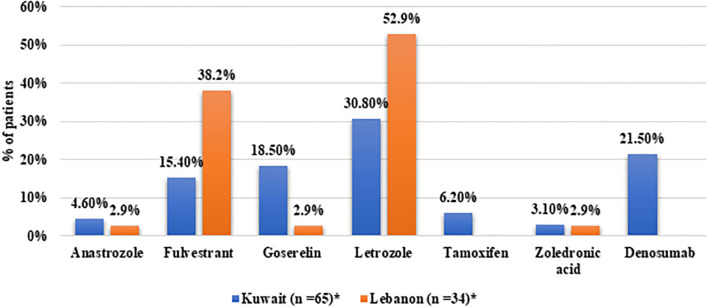
Distribution of medication classes in patients receiving combination therapy from Kuwait and Lebanon. *Patients may have received more than one medication.

In terms of treatment patterns, 56.5% of the patients received abemaciclib in the first-line setting, 19.8% received it in the second-line, and 16.5% received it at third or later lines of treatment. The distribution of the line of abemaciclib treatment was comparable between Kuwait and Lebanon. All patients from Kuwait and 90.7% of Lebanon received abemaciclib 150mg twice daily, while the four patients from Lebanon who were on monotherapy received abemaciclib 200mg (**Table 2**). Regarding combination therapy, ET was the most common combined treatment among patients from Kuwait (75.4%). Letrozole was the most common agent reported to be combined with abemaciclib therapy. Bone-modulating agents were combined with abemaciclib in 24.6% of the patients from Kuwait. On the other hand, letrozole was combined with abemaciclib in 52.9% of the patients from Lebanon, followed by fulvestrant in 38.2% (**Figure 2**).

**Table 2:** Treatment patterns of abemaciclib.

Variables	Total (N=85)	Kuwait (N=42)	Lebanon (N=43)
Line of therapy, No. (%)			
First Line	48 (56.5%)	25 (59.5%)	23 (53.5%)
Second Line	16 (19.8%)	7 (16.7%)	9 (20.9%)
Third Line	14 (16.5%)	6 (14.3%)	8 (18.6%)
Other	7 (8.2%)	4 (9.5%)	3 (7.0%)
Dose at Initiation, No. (%)			
150 mg	81 (95.3%)	42(100%)	39 (90.7%)
200 mg	4 (4.7%)	0 (0%)	4 (9.3%)
Frequency at Initiation, No. (%)			
BID	84 (98.8%)	42 (100%)	42 (97.7%)
Other	1 (1.2%)	0 (0%)	1 (2.3%)
**Dose reduction from starting Abemaciclib to the end of 3 months, No. (%)**	18 (21.4%)	13 (31%)	5 (11.6%)
**Duration of 1^st^ dose reduction, Mean (SD)**	57 (31.3)	57.7 (28.6)	54.0 (49.3)
**Dose of 1st dose reduction, No. (%)**	(n =18)	(n =13)	(n =3)
100 mg	15 (83.3%)	13 (100%)	2 (40.0%)
150 mg	3 (16.7%)	0 (0%)	3 (60.0%)
**Frequency of 1st dose reduction, No. (%)**	**(n =18)**	**(n =13)**	**(n =3)**
BID	15 (83.3%)	13 (100%)	2 (40.0%)
Other	3 (16.7%)	0 (0%)	3 (60.0%)
**Dose reduction since the end of 3 months of Abemaciclib, No. (%)**	**(n = 61)**	**(n =27)**	**(n =34)**
Yes	4 (6.6%)	3 (11.1%)	1 (2.9%)
**Duration of 2^nd^ dose reduction, Mean (SD)**	39.5 (35.2)	42.7 (42.4)	30.0
**Dose of 2^nd^ dose reduction, No. (%)**	**(n =4)**	**(n =3)**	**(n =1)**
50 mg	1 (25%)	1 (33.3%)	0 (0%)
100 mg	3 (75%)	2 (66.7%)	1 (100%)
**Frequency of 1st dose reduction, No. (%)**	**(n =4)**	**(n =3)**	**(n =1)**
BID,	4 (100%)	3 (100%)	1 (100%)
**Radiotherapy since starting Abemaciclib, No. (%)**	4 (4.7%)	2 (4.8%)	2 (4.7%)

**SD**, Standard deviation; **BID**, twice daily.

At the end of the third month of follow-up, 18 patients (21.4%), who were mainly from Kuwait (n =13), had a dose reduction. While at the end of the follow-up, only four patients (6.6%) had a dose reduction; three of them had a dose reduction to 100mg. Four patients (4.7%) received radiotherapy during abemaciclib treatment (**Table 2**).

**Table 3** shows the response rate to abemaciclib. Data regarding the response rate was available for 58 patients at the end of the third month of follow-up. The rates of complete response (CR) and partial response (PR) as the best response were 6.9% and 63.8%, respectively, with an ORR of 70.7%. The rate of CR was comparatively higher in Lebanon (11.8%) than in Kuwait (4.9%); however, patients from Kuwait had a notably higher PR rate (78%) than those from Lebanon (29.4%). Overall, the ORR was higher in Kuwait (82.9%) than in Lebanon (41.2%). Data from 61 patients were available beyond the three months of abemaciclib. The CR and PR rates in those patients were 8.7% and 15.2%, respectively (ORR =23.9%).

**Table 3:** Summary of Best Response (iRECIST 1.1).

Timepoint	Best Response	Total (N=85)	Kuwait (N=42)	Lebanon (N=43)
**2^nd^ timepoint**	Missing	27 (31.8%)	1 (2.4%)	26 (60.5%)
	CR	4 (6.9%)	2 (4.9%)	2 (11.8%)
	PR	37 (63.8%)	32 (78%)	5 (29.4%)
	SD	10 (17.2%)	4 (9.6%)	6 (35.3%)
	PD	7 (12.1%)	3 (7.2%)	4 (23.5%)
	ORR	41 (70.7%)	34 (82.9%)	7 (41.2%)
**3^rd^ timepoint**	n	61	27	34
	Missing	15 (24.6%)	0 (0%)	15 (44.1%)
	CR	4 (8.7%)	1 (3.7%)	3 (15.8%)
	PR	7 (15.2%)	3 (11.1%)	4 (21.1%)
	SD	25 (54.3%)	18 (66.7%)	7 (36.8%)
	PD	10 (21.7%)	5 (18.5%)	5 (26.3%)
	ORR	11 (23.9%)	4 (14.8%)	7 (36.8%)

CR, complete response; PR, partial response; SD, stable disease; **PR**, progressive disease; ORR, objective response rate.

**Figure 3:**
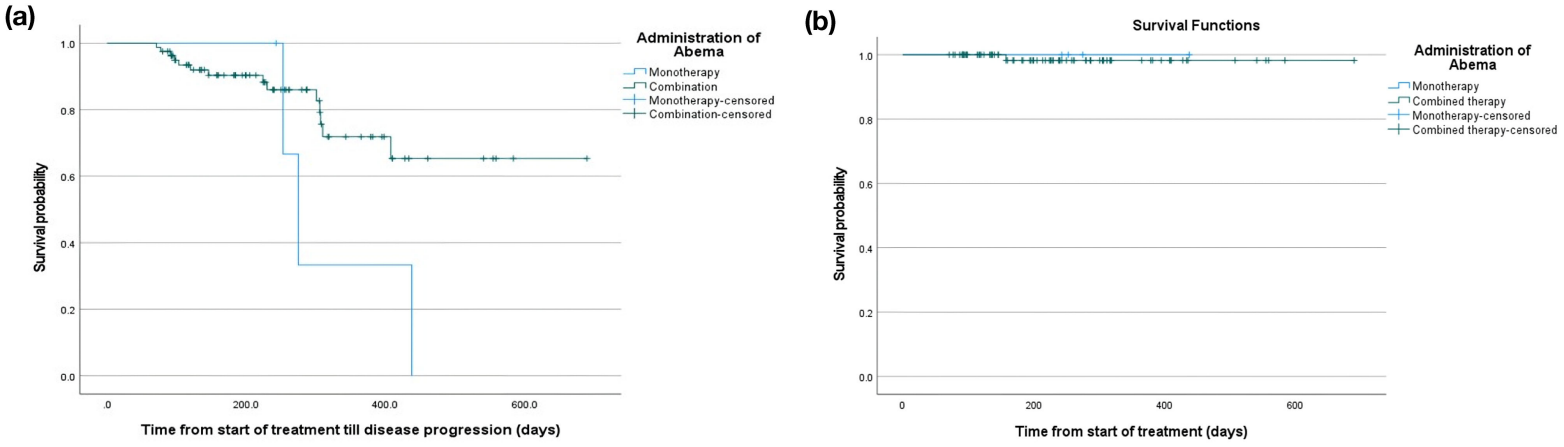
Kaplan Meier curve of **(a)** PFS and **(b)** OS of patients who received abemaciclib monotherapy and in combination.

The median follow-up duration for monotherapy and combination groups were 264 (interquartile range [IQR] 248-356.5) and 226 (IQR 133-308) days, respectively. The median PFS was 275 (95% CI 239.8-310.2) days in the monotherapy group. On the other hand, the median PFS in the combination group was not reached, with a restricted mean PFS of 540.5 (35.5) days. The 12-month PFS was 33.3% in the monotherapy group and 79.6% in the combination group (**Figure 3a**). Concerning OS, one case died in the combination arm; thus, the mean and median time to the event were not assessed. However, the overall follow-up time from the start of treatment showed a median of 264 (IQR 248-356.5) days in the monotherapy group compared to 226 (IQR 136-307) days in the combination group (**Figure 3b**).

We stratified the survival outcomes according to the country. In the Kuwait subgroup, five patients reported disease progression with restricted mean PFS of 563.8 (SE 8.2) days (**Supplementary Figure 1a**). Three progression events in the monotherapy arm versus two in the combination arm were observed in the Lebanon subgroup. The survival analysis showed that the restricted mean PFS in the monotherapy arm was 322.0 (SE 58.3) days versus 498.2 (SE 28.2) days in the combination group (**Supplementary Figure 1b**).

## Discussion

BC is the most prevalent malignancy in Lebanon and Kuwait, with estimated incidence rates of 104.4 and 56.1 per 100,000, respectively, ranking them among the countries with the highest BC incidence in the MENA region (19, 21). Previous epidemiological studies showed that nearly 36.4% and 28% of BC patients from Lebanon and Kuwait present with stage III-IV BC at the time of diagnosis, respectively (25, 26). Despite the considerable burden of advanced disease in both countries, limited real-world data are available regarding the treatment patterns and outcomes of CDK4/6 inhibitors. Abemaciclib has recently gained approval in Lebanon and Kuwait in first and second-line advanced BC settings. The present real-world study evaluated the treatment patterns and survival outcomes of abemaciclib plus ET or as a single agent in patients with HR+/HER2- locally advanced or mBC in Kuwait and Lebanon.

Abemaciclib is an orally administered CDK4/6 inhibitor that is dosed on a twice-daily continuous schedule. The recommended starting dose in combination with ET is 150 mg twice daily; however, the recommended starting dose as monotherapy is 200 mg twice daily (27). In locally advanced or metastatic settings, abemaciclib is approved in combination with aromatase inhibitors (AI) in the first-line setting or at later lines as monotherapy following progression on ET and prior chemotherapy. The current international guidelines recommend the early introduction of CDK4/6 inhibitors, including abemaciclib, in combination with ET for HR+/HER2- locally advanced or mBC (28, 29). However, previous real-world studies noted heterogeneity between guideline recommendations and drug use during the early post-approval period (30, 31).

Nonetheless, the present real-world evidence showed that the treatment patterns at the time of starting abemaciclib align with international recommendations. The majority of the patients in Kuwait and Lebanon received abemaciclib 150mg twice daily in combination with AI as first-line therapy, while findings from two recent real-world studies from the United States (US) showing a trend towards abemaciclib initiation in patients with features of advanced stages such as later lines of treatment, prior ET or CKD 4/6 inhibitors, visceral metastases, and poor ECOG status (32, 33). The authors hypothesized these findings are attributed to the physicians’ tendency to prescribe abemaciclib once it was approved for patients with a longer duration of metastatic disease or prior lines of therapy. Besides, it should be noted that abemaciclib received the frontline indication in February 2018, while the data collection window for the two studies from the US covered the period from June 2016/September 2017 to December 2018/October 2019 (32, 33).

The survival benefits of abemaciclib have been demonstrated as a frontline therapy (15), in combination with fulvestrant in women who progressed on ET (34). Such findings were further observed in a real-life setting (32, 33). In the present study, the ORR was 70.7% after three months of abemaciclib treatment; the rate of ORR was higher in Kuwait (82.9%) than in Lebanon (41.2%), which can be explained by the higher percentage of patients with prior lines of therapy and the large number of patients with missing RECIST response in Lebanon cohort. The 12-month PFS was 33.3% in the monotherapy group and 79.6% in the combination group. Such findings confirm that the benefits of abemaciclib observed in clinical trials are reflected in the real-world setting; real-world data usually encompasses a larger pool of patients with diverse features and less strict criteria than those in clinical trials. In the MONARCH 3 trial, the ORR of frontline abemaciclib was 61%, and the median PFS was 28.18 months (35). Likewise, the ORR was 35.2% in women who progressed on ET in MONARCH 2 (36). The real-world response rate and survival benefits in the present study were notably higher than those reported in other real-world evidence from the US. In Carter et al., the ORR was 41.2%, and the 12-month PFS was 61% (33). However, as highlighted above, nearly two-thirds of the patients in Carter et al. received abemaciclib in a more advanced stage than our cohort. Besides, the assessment of tumor response in Carter et al. was based on the physician’s assessment rather than the RECIST criteria used in the present study.

In MONARCH trials, abemaciclib was associated with manageable safety profiles and a low incidence of severe adverse events leading to treatment discontinuation; neutropenia, diarrhea, and leukopenia were the most common adverse events in MONARCH trials (35). Although the present real-world study did not capture the incidence of adverse events, we found that the rate of dose reduction, as a measure of treatment tolerability, was 21.4% within the first three months of abemaciclib treatment. The mean duration of dose reduction was 57 ± 31.3 days. In a real-world study from the US, the rate of dose reduction in mBC patients receiving abemaciclib was 30.8% (32). These rates are also consistent with the real-world evidence evaluating other CKD 4/6 inhibitors, palbociclib, and ribociclib, in patients with mBC (37–39). The rate of dose reduction in the present study was lower than the reported figures from MONARCH 2 and 3 trials, 42.9% and 43.4%, respectively (40). Such heterogeneity is expected due to the strict dosing protocols followed in the clinical trials, which are usually lacking in the real-world setting. Nonetheless, the impact of dose reduction on the outcomes of abemaciclib appears to be insignificant, as recently reported in a subgroup analysis of MONARCH 2 and MONARCH 3 data (40).

### Limitations

To our knowledge, the present study is the first real-world evidence to investigate the treatment patterns and survival outcomes of CKD 4/6 inhibitors in women with HR+/HER2- locally advanced or mBC in the MENA. However, we acknowledge that the study has certain limitations. The retrospective nature of the study can increase the risk of misclassification and recall bias. The sample size was relatively small, and the median duration of follow-up was limited, as the study was initiated within one year of the approval of abemaciclib in Kuwait and Lebanon. The small sample size and short follow-up duration limited the maturity of the time-to-event analyses and the feasibility of conducting subgroup analysis according to the treatment regimen (monotherapy versus combination). Data regarding the response rates and survival were not available for all patients, especially the cohort from Lebanon. As a real-world retrospective study, PFS was not assessed through standardized, prospective serial imaging. Instead, radiographic progression was determined based on the available imaging studies conducted as part of routine clinical care, which may have introduced variability in assessment intervals across patients. The timing and frequency of imaging were not protocol-driven, and there may have been inconsistencies in follow-up imaging due to variations in clinical practice, patient condition, or physician discretion. Furthermore, data were not available to stratify PFS according to the line of treatment. The present study also did not capture the toxicity profile of abemaciclib. Although we have collected several important variables, we acknowledge that, due to the retrospective nature of the study, our dataset was limited to the routinely monitored data in real-world practice. Future prospective studies from the Middle East, with larger sample sizes and longer follow-ups, are needed to understand the toxicity profile of abemaciclib and provide a more comprehensive analysis of the region.

## Conclusion

The present real-world evidence confirms the feasibility and effectiveness, in terms of response rate and PFS, of abemaciclib in patients with HR+/HER2- patients with locally advanced or mBC from Kuwait and Lebanon. In both countries, abemaciclib was usually used in the frontline setting in combination with AI or as a part of a combination regimen for women who progressed on ET. The observed real-world effectiveness in the present study was comparable to the results of pivotal MONARCH trials, further complementing clinical trial results. Further research is warranted to compare the real-world response and survival outcomes of abemaciclib according to the line of therapy.

## Data Availability

As this is an observational study that uses data previously collected and does not impose any form of intervention, the data has been de-identified to protect subject privacy. Therefore, a formal consent to release information form was not required, and a waiver of informed consent was requested from the IRB/IEC by the PI. Requests to access these datasets should be directed to rosca_alexandru@lilly.com.
