# Supplementary figures and images for: Abemaciclib treatment patterns and outcome in HR+/HER2- locally advanced or metastatic breast cancer: a real-world study from Kuwait and Lebanon
Source: Front Oncol. 2025 Jun 17;15:1437380. doi: 10.3389/fonc.2025.1437380 (PMC12210239; doi:10.3389/fonc.2025.1437380)

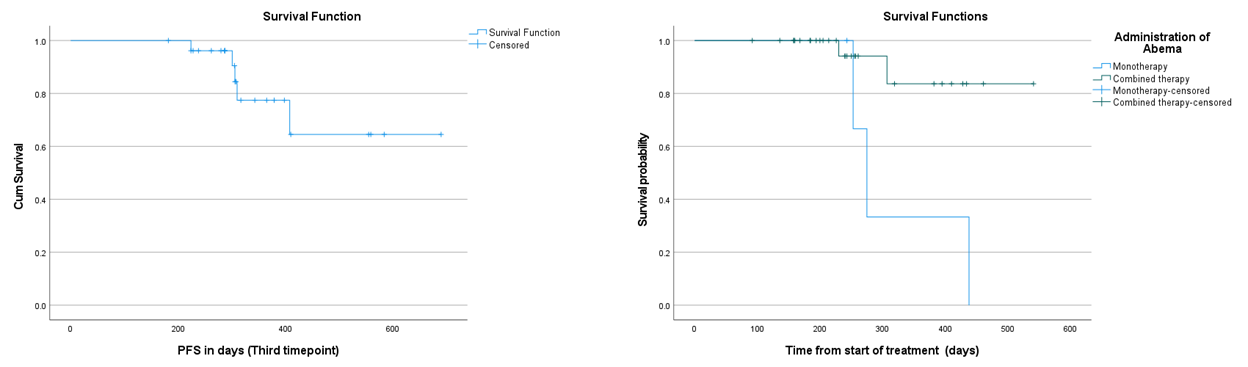

Supplement: Supplementary file 1 [file Image1.png]
